# Resin Infiltration for the Esthetic Improvement of Dental Fluorosis and White Spots: A Case Report

**DOI:** 10.7759/cureus.72493

**Published:** 2024-10-27

**Authors:** Sumayyah L Alkhudhayri, Shahad L Alhassani, Nada A AbdelAleem

**Affiliations:** 1 Dentistry, Security Forces Hospital, Makkah, SAU; 2 Dentistry, Heraa General Hospital, Makkah, SAU; 3 Restorative Dentistry, Faculty of Dental Medicine, Umm Al-Qura University, Makkah, SAU

**Keywords:** dental fluorosis, icon resin infiltrant, initial caries, resin infiltration, white spot lesion

## Abstract

White spot lesions, characterized by opaque enamel discolorations from early carious lesions, fluorosis, or developmental issues, can be effectively managed through various treatments. Resin infiltration is a unique therapy that bridges the gap between non-invasive and invasive alternatives, potentially delaying the need for restorations by applying a low-viscosity resin that penetrates and seals the lesion, enhancing both appearance and function. This study evaluates the clinical and esthetic outcomes of resin infiltration in a 33-year-old female patient with mild dental fluorosis, who presented with white and brown discolorations on her anterior teeth and a high caries index. Following a comprehensive treatment plan that included scaling, root planning, and resin infiltration, several diagnostic tests - such as Endo-Ice, Electric pulp testing, DIAGNOdent, and Spectrophotometer - were utilized to assess treatment success, showing reduced pulp sensory response times, improved caries detection values, and noticeable esthetic enhancements. However, one-month post-treatment, the patient reported yellow staining on the treated surfaces due to turmeric consumption, necessitating additional finishing and polishing. This case underscores the effectiveness of resin infiltration in improving both esthetic and clinical outcomes for fluorosis and incipient carious lesions, emphasizing the importance of patient education regarding dietary habits and oral hygiene to prevent staining and ensure long-term success, while further research is warranted to explore the long-term efficacy of resin infiltration and develop strategies to mitigate post-treatment discoloration.

## Introduction

White spot lesions are enamel lesions that appear opaque and chalky white. It may occur from an idiopathic source, an early caries lesion, or a developmental reason like fluorosis [1]. Dental fluorosis is a condition that causes white or yellowish lesions on the hard tissues of the mouth. The literature suggests several treatments, some of those being even invasive. In terms of the resolution of lesions and patient satisfaction over time, the ICON resin infiltration technique was discovered to be successful in the consistent resolution of lesions. The treatment uses a low-viscosity resin designed to penetrate and seal areas of demineralized enamel. This helps to stop the progression of caries and improve the esthetic appearance of the teeth, especially in cases of white spots, which are often a result of demineralization [1].

Another type of white spot lesion is enamel caries (incipient caries), which can be diagnosed clinically for the first time. A dietary carbohydrate and saliva-modified bacterial infection that causes white spot lesions results from an imbalance between the demineralization and remineralization of the enamel [2]. White spot lesions are frequently found and can be stressful for patients with strong esthetic concerns, several approaches have been proposed for the management of white spots and fluorosis lesions. These include the use of therapeutic sealants for occlusal lesions or the remineralization of the lesion with fluoride and casein phosphopeptide amorphous calcium phosphate [3]. Another non-invasive alternative treatment has been developed for the management of non-cavitated caries lesions, it has been introduced by Kielbassa et al. [4]. The caries infiltration is a unique therapy option for these lesions that may fill the "gap" between non-invasive and invasive alternatives, potentially delaying the first placement of a restoration. The purpose of caries infiltration is to use a low-viscosity resin (infiltrate) to soak up the porous lesion body and then harden it with blue light. By doing this, lesions are sealed, and cariogenic acid diffusion channels are inhibited [5].

## Case presentation

Clinical presentation

**Figure 1:**
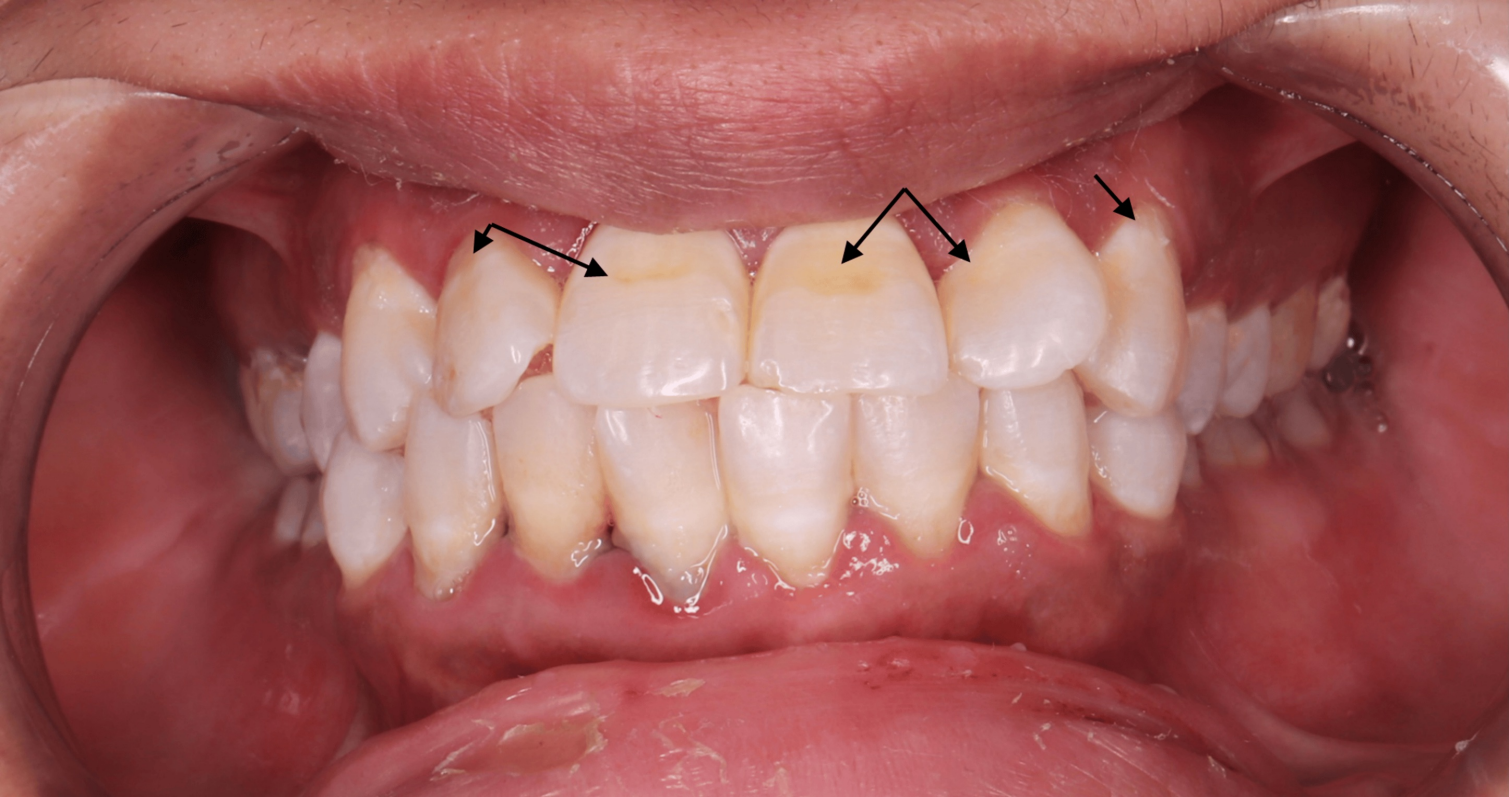
Baseline of white and brown lesions in upper anterior teeth.

A 33-year-old female patient sought dental care with a chief complaint of white and brown discolorations on her anterior teeth. She had been diagnosed with rheumatoid arthritis eight years ago but was not on significant medication at the time of presentation. On extraoral examination, there were no signs of lymphadenopathy or facial asymmetry, though temporomandibular joint assessment revealed clicking sounds and pain during mouth movements. Intraoral examination revealed white spots and brown stains on the labial surfaces of her upper anterior teeth, consistent with a mild form of dental fluorosis according to Dean’s 1942 classification (Figure 1). Additionally, the patient had a high caries index according to the American Dental Association’s assessment. After discussing the various treatment options and potential complications, a comprehensive treatment plan was established to address her oral health concerns.

Treatment procedure

The treatment started with scaling and root planning, along with oral hygiene instructions and patient motivation. Resin infiltration (RI) was then performed under partial isolation with cotton rolls to avoid contamination and soft tissue injury. The teeth were cleaned with prophylaxis paste and polishing brushes before the RI procedure. The enamel was etched with 15% hydrochloric acid (Icon Etch) for 120 s, then rinsed for 30 s and dried. Followed with 99% pure ethanol (Icon Dry) for 30 seconds. This cycle repeated three times. The Icon infiltration resin was then applied to the tooth surfaces for 3 min, the excess resin was removed using dental floss, and photopolymerization was conducted with a light-curing unit (LCU). The LCU tip was positioned parallel to the teeth at 1 mm for 40 s. A second layer of Icon infiltration was applied and cured for 40 s. Finally finishing was done using interproximal strips and finishing discs, followed by polishing with a paste.

Treatment outcome

**Figure 2:**
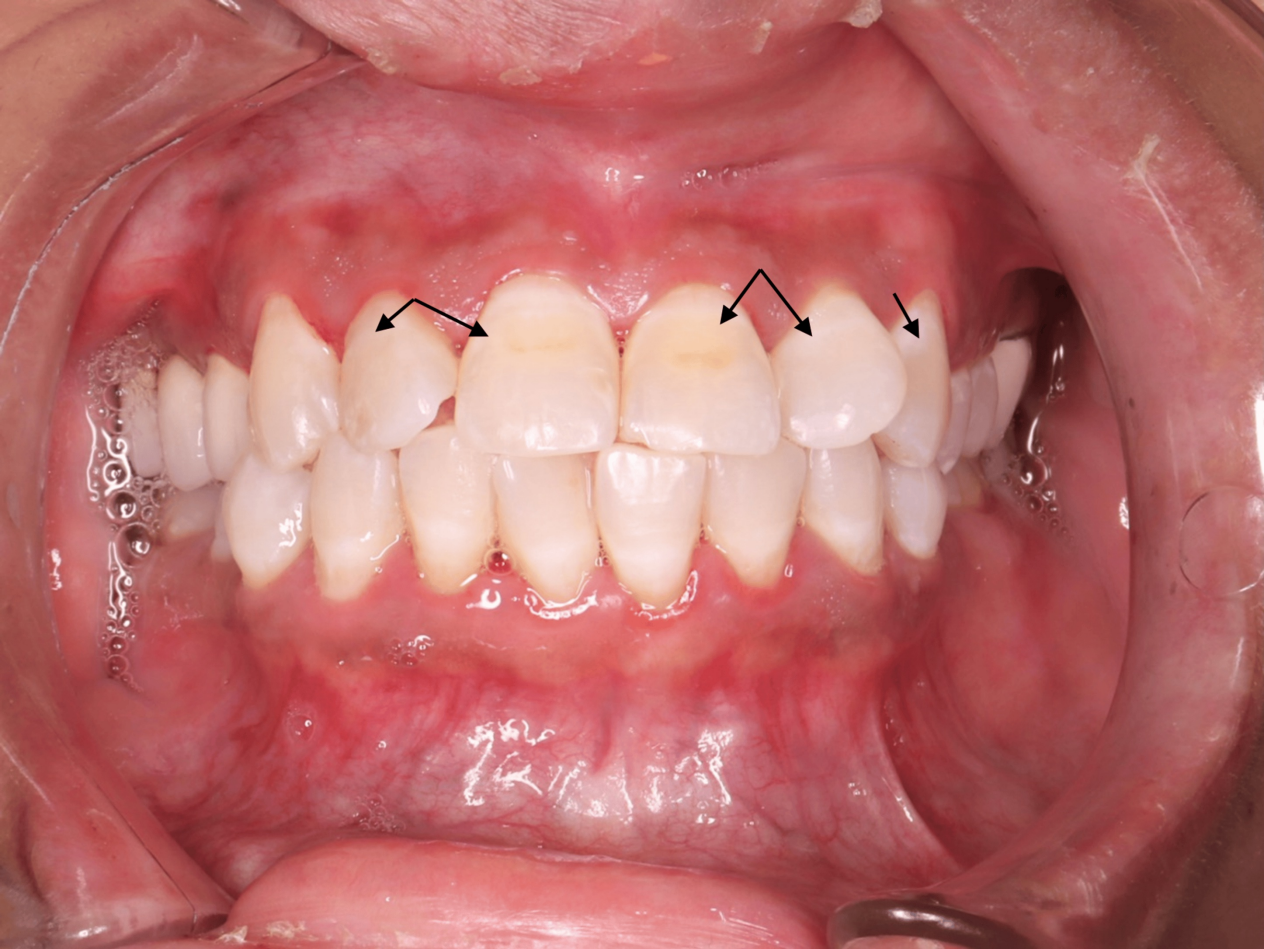
One week follow-up after RI of white and brown lesions in upper anterior teeth. RI: resin infiltration

To evaluate the effects of the resin infiltration, several tests were conducted before and after the procedure (Table 1). The Endo-Ice test, which measures pulp sensory response in seconds, revealed a decrease in response times post-RI, with notable reductions for teeth 6, 7, 8, and 11. However, tooth 10 showed an increase in response time from 0 to 3.1 s. The Electric pulp test, measuring sensory response in microamperes (μA), demonstrated a significant increase in pulp sensitivity after RI, with readings rising from 3 to 24 μA before treatment to 23 to 64 μA afterward, particularly in teeth 10 and 11. The DIAGNOdent test, used to detect carious lesions, showed a reduction in caries detection values for most teeth post-RI, indicating an improvement, although an increase was observed for tooth 9. Finally, spectrophotometer analysis of tooth color codes revealed changes before and after RI, indicating alterations in tooth appearance, such as tooth 6 changing from 2M2, A3 to 2R2.5, A3, and tooth 10 shifting from 2L1.5, C2 to 2R2.5, A1. Post-treatment evaluations showed positive outcomes, including good gingival health and a slight improvement in brown lesions after one week (Figure 2).

**Table 1:** Different parameters to assess the teeth before and after resin infiltration.

Teeth	Endo-Ice test (S)		Electric pulp testing (μA)		DIAGNOdent		Spectrophotometer	
	Before	After	Before	After	Before	After	Before	After
6	2.25	1.5	10	23	1	1	2M2, A3	2R2.5, A3
7	4.2	1.4	24	52	1	0	2R2.5, A3	2M1, A1
8	1.77	1.5	22	64	0	0	2M1c, C1	2M1, A1
9	2.1	2.1	4	64	0	1	4R1.5, D2	2R1.5, A2
10	0	3.1	3	64	3	1	2L1.5, C2	2R2.5, A1
11	5.1	1.4	17	64	1	1	2R2.5, A2	2R1.5, A2

**Figure 3:**
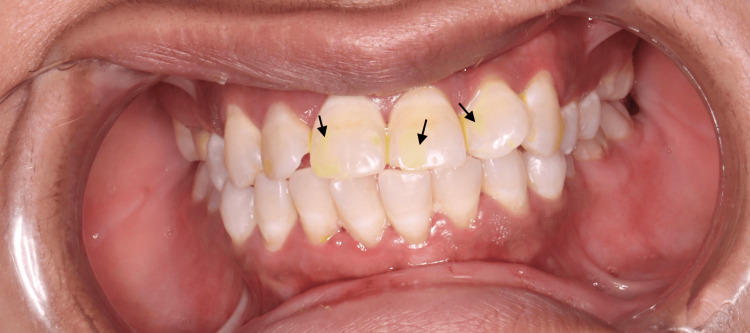
One month follow-up after RI of white and brown lesions in upper anterior teeth, there was yellow discoloration. RI: resin infiltration

**Figure 4:**
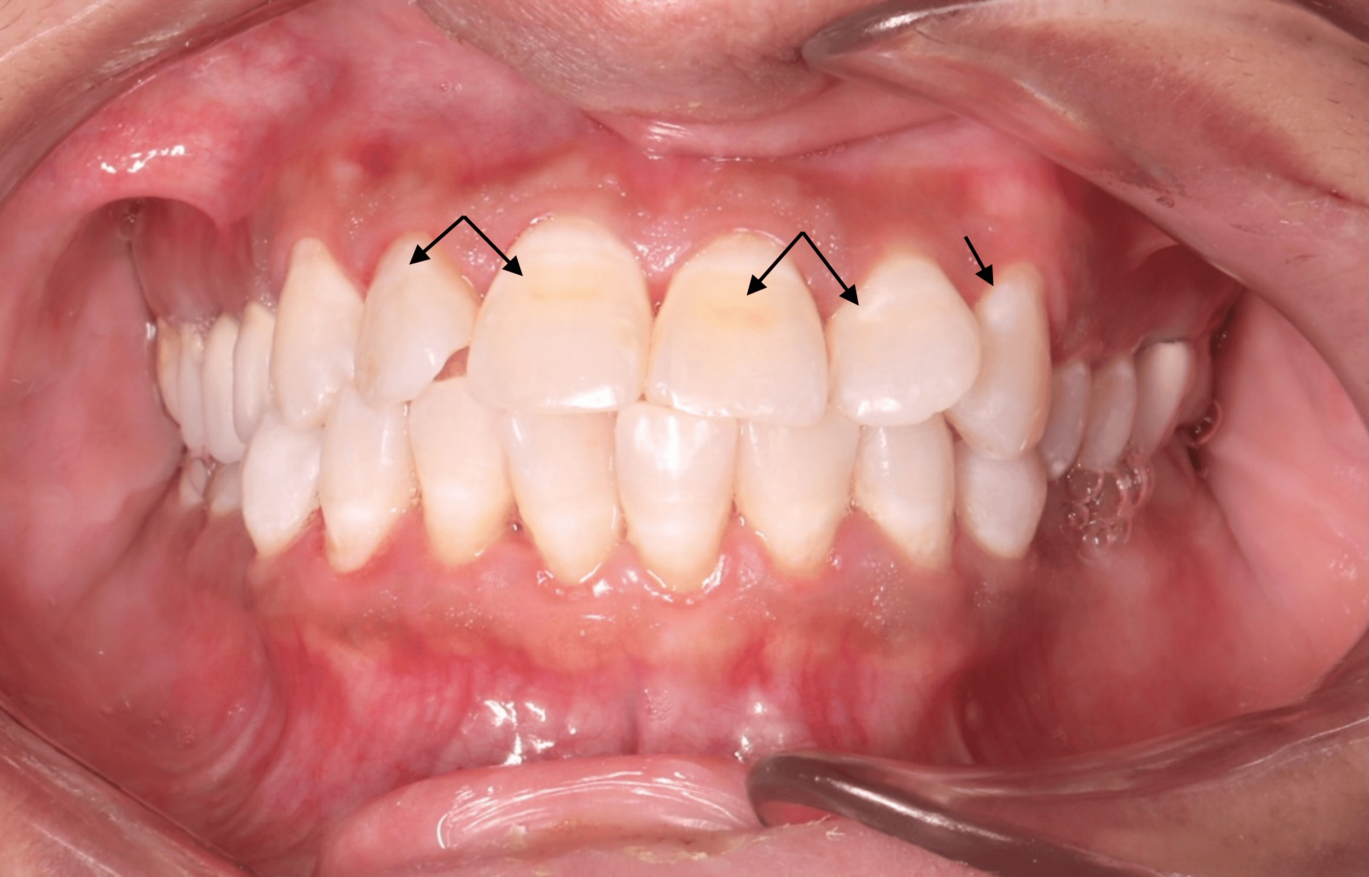
One year follow-up after RI of white and brown lesions in upper anterior teeth. RI: resin infiltration

However, after one month, the patient reported yellow discoloration on a tooth affected by turmeric staining (Figure 3). This issue was addressed by performing additional finishing with discs and polishing with paste. The patient reported experiencing yellow discoloration on their teeth a year after consuming staining foods, such as turmeric. The discoloration persisted for a few days but disappeared after discontinuing the staining foods and brushing twice daily with a whitening toothpaste (Figure 4). These findings underscore the efficacy of resin infiltration for esthetic and clinical enhancement, while also emphasizing the need for ongoing patient education and care to manage potential issues such as staining. Further research is warranted to explore the long-term effects and refine the application of this treatment.

## Discussion

The case presented a clear clinical application of the resin infiltration (RI) technique for managing white spot lesions, particularly in patients with dental fluorosis. Initially, the patient reported esthetic concerns with white and brown discolorations on her anterior teeth, which were diagnosed as mild dental fluorosis according to Dean's 1942 classification. The treatment strategy selected resin infiltration was aimed not only at improving the esthetics but also at preserving the vitality of the teeth. This is particularly crucial, as the treatment involved non-invasive methods to address the lesions, avoiding the need for more invasive procedures such as restorations or enamel reductions.

When comparing the results before and after the resin infiltration procedure, significant improvements were observed. The Endo-Ice test, which measures the pulp sensory response, showed a notable reduction in response times after treatment. For instance, teeth 6, 7, 8, and 11 exhibited shorter sensory response times post-treatment, indicating an improvement in pulp vitality. However, tooth 10 was an exception, showing an increase in response time, suggesting a different level of sensitivity or a possible minor complication during the treatment.

Similarly, the Electric pulp test results demonstrated an increase in the responding time, which indicates a decrease in sensitivity after RI, particularly in teeth 10 and 11, where the measurements rise from 3 mA to 64 mA, indicating a decrease in the sensory function and pulp viability. This suggests that the resin infiltration not only addressed the esthetic concerns but also had a positive impact on pulp health, possibly by sealing the lesion and reducing the exposure to cariogenic factors.

The DIAGNOdent readings, which assess caries detection, also showed a general reduction post-treatment. Most of the teeth exhibited lower caries detection values, indicating an improvement in the condition of the enamel. The exception was tooth 9, which showed a slight increase, pointing to the need for continuous monitoring of this specific tooth.

In terms of esthetics, the spectrophotometer analysis of tooth color revealed significant changes in the tooth shade codes. For example, tooth 6 changed from a shade of 2M2, A3 to 2R2.5, A3, and tooth 10 shifted from 2L1.5, C2 to 2R2.5, A1, indicating improvements in both brightness and color uniformity. These results align with the patient’s initial concerns, showing that the resin infiltration procedure was successful in addressing the discolorations.

This study focused on treating mild dental fluorosis using resin infiltration (Icon), which significantly improved esthetic issues and detection values (DIAGNOdent readings). However, after one month, the patient experienced turmeric-induced staining, requiring additional finishing.

Studies by Divyameena et al. and Saxena et al. explored the combination of enamel microabrasion and resin infiltration for fluorosis treatment, both aiming to improve the appearance of stains and reduce demineralization [6,7]. This study, however, focused on resin infiltration, suggesting the potential for improved outcomes when combining techniques.

Borges et al. examined resin infiltration’s color stability, highlighting that while it is effective, it remains susceptible to staining from dietary sources, similar to our finding with turmeric [8]. Zhao and Ren also emphasized that diet plays a key role in maintaining esthetic results [9]. Paris et al. confirmed that resin infiltration effectively seals lesions and prevents caries progression, aligning with our findings on its esthetic benefits and protective qualities [5]. Overall, our study supports the effectiveness of resin infiltration but highlights the importance of managing staining through patient education on diet and maintenance.

## Conclusions

The resin infiltration technique proves to be an effective, minimally invasive approach for enhancing the esthetics and clinical outcomes of dental fluorosis and white spot lesions. This case highlights significant improvements in both tooth vitality and appearance, reinforcing the technique's role in modern dental treatment.

While resin infiltration successfully addresses esthetic concerns, it is crucial for patients to maintain good oral hygiene and be mindful of dietary choices that may lead to staining. Continuous patient education on these factors is essential for ensuring long-term success and satisfaction with the treatment outcomes. Further research is needed to explore the durability of the results and strategies to mitigate potential staining from everyday foods.
